# The Epidemiology of Disability in Saudi Arabia: National Prevalence, Functional Multimorbidity, and Age-Stratified Self-Reported Causes

**DOI:** 10.3390/jcm15197504

**Published:** 2026-09-27

**Authors:** Saad M. Bindawas, Vishal Vennu

**Affiliations:** 1 Department of Rehabilitation Sciences, College of Applied Medical Sciences, King Saud University, P.O. Box 10219, Riyadh 11433, Saudi Arabia; vvennu@ksu.edu.sa; 2 King Salman Center for Disability Research, P.O. Box 94682, Riyadh 11614, Saudi Arabia

**Keywords:** disability, functional difficulty, Washington Group, Saudi Arabia, functional multimorbidity, regional distribution, disability inclusion, population statistics

## Abstract

**Background/Objectives**: Disability reflects interactions between health and contextual factors. Washington Group (WG) measures capture functional difficulty but not disabling barriers. Thus, this study aimed to describe national disability patterns in Saudi Arabia using aggregate WG-aligned indicators. **Methods**: We conducted a descriptive cross-sectional secondary analysis of aggregate data from the Saudi Population and Housing Census 2022 and the 2023 Disability Survey published by the General Authority for Statistics. The 2023 survey covered 29,920 households across 13 administrative regions and individuals aged ≥2 years. Disability was operationalized as severe difficulty or inability in at least one functional domain (WG Grades 2–3). National counts were derived from published proportions and Census 2022 denominators. **Results**: Combined disability and functional difficulty affected 4.2% of the population, corresponding to approximately 1.35 million people. Applying published Grade 2–3 proportions to Census 2022 denominators yielded an estimated 587,900 people meeting the operational disability threshold. Multiple functional domains were reported among 58.5% of severe-or-unable cases, increasing to 68.2% among children aged 2–4 years. Grade 2–3 prevalence was 1.4% among adults aged 15–64 years and 15.1% among those aged ≥65 years. Illness was the most frequently reported reason among adults aged ≥18 years (44.2%) and overall (39.2%). Ar-Riyadh, Makkah, and the Eastern Region accounted for 44.0% of severe-or-unable single-disability cases. **Conclusions**: Disability in Saudi Arabia transitions from developmental and multimorbid issues in childhood to illness-related disabilities in adulthood. These findings support disability-inclusive health planning and rehabilitation strategies under Saudi Vision 2030.

## 1. Introduction

Disability is both a population-health and human-rights concern. The International Classification of Functioning, Disability and Health (ICF) conceptualizes functioning and disability in relation to health conditions and contextual factors, including environmental and personal factors that influence activity and participation [1,2]. Contemporary rights-based approaches, including the United Nations Convention on the Rights of Persons with Disabilities (CRPD), further emphasize equality, accessibility, participation, inclusion, and freedom from discrimination [3,4]. From this perspective, congenital conditions, illness, aging, and functional difficulties may be associated with impairment or limitations in functioning. Still, they do not by themselves explain the social experience of disability.

The Washington Group (WG) has developed standardized questions for population-based measurement of functional difficulty and disability, supporting internationally comparable statistics and monitoring relevant to the CRPD and Sustainable Development Goals [5,6]. These measures help identify people who experience substantial difficulty in core functional domains. At the same time, a WG-based statistical threshold is an operational measure for population surveillance; it does not capture all dimensions of disability, including environmental accessibility, discrimination, institutional barriers, social participation, or availability of supports.

Saudi Arabia is undergoing demographic and epidemiological change, including population aging and a substantial prevalence of chronic health conditions [7,8]. These developments may influence patterns of functional difficulty. At the same time, the extent to which people experience disability in everyday life also depends on accessibility, social and institutional arrangements, and access to supports and services. Previous national estimates used different measurement approaches, limiting direct comparison across periods [8,9,10].

The 2022 Population and Housing Census and the 2023 Disability Survey provide recent national indicators using WG-aligned measures. The present study does not treat these sources as an individually linked dataset; instead, it synthesizes their published aggregate indicators to provide a transparent descriptive profile of prevalence, functional multimorbidity, regional composition, and self-reported reasons. The objective was to characterize the published national indicators and identify questions requiring individual-level, age-standardized, longitudinal, and rights-oriented research. We did not estimate causal determinants, comparative regional risks, or verified causal mechanisms.

## 2. Materials and Methods

### 2.1. Study Design and Setting

We conducted a descriptive, cross-sectional secondary analysis of national aggregate indicators derived from two public sources: the Saudi Population and Housing Census 2022 and the 2023 Disability Survey, published by the General Authority for Statistics (GASTAT), Kingdom of Saudi Arabia [11]. Covering all 13 administrative regions, these datasets are described as ‘combined sources’ to emphasize that they represent unlinked aggregate statistics without access to individual microdata. This study adheres to the STROBE reporting framework and its RECORD extension for observational analyses of routinely published statistics (Table S1) [12].

### 2.2. Participants and Data Sources

The 2023 Disability Survey covered 29,920 Saudi and non-Saudi households with residents aged ≥2 years across all administrative regions. GASTAT reports that the survey used computer-assisted personal interviewing and a sample drawn from the 2022 Census frame. The Census 2022 provided national prevalence indicators, while the 2023 Disability Survey provided aggregate indicators on severity, multiplicity, self-reported reasons, regional household indicators, and other characteristics. The present analysis used only the published aggregate outputs; individual records, sampling weights, standard errors, and respondent-level covariates were not available to the authors.

### 2.3. Disability Measurement and Conceptual Scope

The 2023 survey used the UNICEF/WG Child Functioning Module for children aged 2–17 years and WG Extended Questions for adults aged ≥18 years [13,14]. GASTAT reports three difficulty grades: Grade 1 (mild), Grade 2 (severe), and Grade 3 (very severe or unable). In the present statistical analysis, the published Grade 2–3 category is referred to as the operational disability threshold, while Grade 1 is referred to as mild functional difficulty [13,15,16]. This terminology describes the measurement convention and should not be interpreted as a complete definition of disability under the CRPD. In particular, the aggregate data do not permit direct measurement of environmental barriers, discrimination, participation restrictions, or the adequacy of supports.

#### Conceptual Scope and ICF Alignment

In accordance with the WHO ICF bio-psycho-social framework, disability arises from the dynamic interaction between health conditions and contextual environmental/attitudinal barriers. The WG operational indicators deployed in the 2023 Survey measure specific functional task difficulties (capacity/performance limitations in core domains). They do not directly measure environmental accessibility, physical infrastructure, institutional policies, or social discrimination. Accordingly, population counts meeting WG Grade 2–3 thresholds represent operational surveillance metrics for service screening rather than exhaustive measures of societal disability under the United Nations CRPD.

### 2.4. Statistical Analysis

We conducted a descriptive secondary analysis summarizing published prevalence proportions across demographic (age, sex, nationality), geographic (13 administrative regions), and clinical (severity, multiplicity, self-reported reasons) strata. To establish population magnitude, derived national counts were computed by applying published prevalence proportions to official Census 2022 denominators (32,175,224 total; 18,803,176 Saudi; 13,372,048 non-Saudi) and rounding to the nearest hundred. These approximations represent transparent aggregate calculations without individual microdata reconstruction, ensuring end-to-end auditability via Supplementary File S1 and Table S2 in accordance with the RECORD statement.

Functional multimorbidity was defined as multiple co-occurring functional difficulties among severe-or-unable cases (WG Grades 2–3). Regional metrics were evaluated under strict denominator separation: household indicators represent household-level prevalence, whereas survey regional compositional shares describe the national distribution of observed cases influenced by regional population mass. Self-reported reasons reflect respondent attributions rather than clinically validated etiological mechanisms, and marginal age/sex distributions were analyzed independently due to the absence of joint microdata cross-tabulations.

Given the absence of individual microdata, sampling weights, and standard errors in public releases, inferential testing, multivariable modeling, interaction analyses, and regional age standardization were omitted to prevent ecological fallacies and false precision. Calculations and data visualizations were executed using Microsoft Excel (Microsoft Corporation, Redmond, WA, USA) and Python (version 3.12.0; Python Software Foundation, Wilmington, DE, USA; https://www.python.org) utilizing pandas (version 2.2.0) and matplotlib (version 3.8.0).

## 3. Results

### 3.1. National Prevalence and Functional Difficulty

Census 2022 indicators reported disability and functional difficulty together in 4.2% of the resident population, corresponding to approximately 1.35 million people. The published prevalence was 5.9% among Saudi citizens and 1.8% among non-Saudi residents. Using the published Grade 2–3 prevalence proportions of 2.7% among Saudi citizens and 0.6% among non-Saudi residents, application of Census 2022 population denominators yielded an estimated 587,900 residents meeting the operational disability threshold (Table 1). These are derived national counts and should be interpreted as approximations.

### 3.2. Functional Multimorbidity

Among severe-or-unable cases in the 2023 Disability Survey, 58.5% were reported as having multiple disability domains. The corresponding proportion was 68.2% among children aged 2–4 years, 60.0% among those aged 5–17 years, and 58.0% among adults aged ≥18 years (Table 2 and Figure 1). Among severe-or-unable cases, 55.0% of males and 59.4% of females reported multiple domains. These percentages describe multiplicity among the surveyed population and do not identify causal determinants of multiple disability domains.

### 3.3. Regional Distribution

Regional variations in Table 3 and Figure 2 should be interpreted with caution. The reported household proportions and regional compositional shares represent raw, unadjusted descriptive metrics. Because the published operational disability prevalence rises from 1.4% among adults aged 15–64 years to 15.1% among adults aged ≥65 years, inter-regional differences may partly reflect variations in regional age demographics rather than underlying differences in health risk or health-system performance. No age-standardized regional comparison was possible from the aggregate indicators used in this study.

We interpreted regional indicators separately because they represent different units and denominators. GASTAT’s household indicator reports the percentage of households with at least one person with a disability, including mild difficulty. The reported values were 14.7% in Ar-Riyadh, 15.0% in Makkah, and 11.7% in the Eastern Region, with lower values in the remaining regions (Table 3; Figure 2A). This indicator is a household-level descriptive measure and should not be interpreted as an age-adjusted regional prevalence.

The 2023 Disability Survey also reported regional compositional shares among severe-or-unable single-disability cases. Ar-Riyadh (14.8%), Makkah (14.7%), and the Eastern Region (14.5%) together accounted for 44.0% of these cases (Figure 2B). These regional case distributions describe absolute or compositional volume rather than regional prevalence. Because the percentages are unadjusted for regional population size and age structure, interpret them as distributions of observed cases, not as evidence of elevated baseline epidemiological risk. These crude volume indicators cannot support inferences about comparative regional healthcare performance or geographic causal patterns.

### 3.4. Self-Reported Reasons and Age/Sex Patterning

Self-reported reasons varied by age group (Table 4; Figure 3). Among children aged 2–4 years, congenital factors (52.4%), pregnancy-related factors (7.0%), and delivery-related factors (30.6%) together accounted for 90.0% of reported reasons. Among adults aged ≥18 years, illness was the most frequently reported reason (44.2%), followed by congenital factors (21.0%) and aging (20.2%). In the total population, illness accounted for 39.2% of reported reasons. These are self-reported or reported-attribution categories in the GASTAT survey and should not be interpreted as independently verified causes of disability.

Traffic accidents represented 7.2% of reported reasons among males and 1.7% among females. The aggregate data do not allow adjustment for age, exposure, occupation, driving patterns, or other potential confounders; therefore, these differences are descriptive and do not establish why the distributions differ by sex.

## 4. Discussion

This study provides a transparent descriptive synthesis of recent GASTAT indicators on disability and functional difficulty in Saudi Arabia. Its principal contribution is to place several published national measures in one analytic framework while explicitly preserving the boundaries of the underlying data. The Census and Disability Survey were not individually linked, and the analysis did not have access to respondent-level covariates, sampling weights suitable for re-analysis, or standard errors. The results therefore describe published distributions rather than associations, causal effects, or adjusted comparative risks.

The 4.2% estimate is the Census indicator combining disability and functional difficulty, whereas the approximately 587,900 estimate applies the published Grade 2–3 proportions to Census 2022 population denominators. These quantities answer different descriptive questions and should not be treated as interchangeable measures of the prevalence of disability under the ICF or CRPD. The WG threshold is useful for standardized population surveillance. Still, it captures only selected aspects of functional difficulty, not the full range of environmental, institutional, and attitudinal factors that shape disability in everyday life.

The reported 58.5% proportion of multiple functional domains among severe-or-unable cases indicates that many surveyed individuals experienced difficulty in more than one domain. The higher proportion among children aged 2–4 years is a descriptive finding that may inform coordination across health, developmental, education, family, and community services. It should not be interpreted as evidence of a specific biological syndrome or as a direct measure of individual burden [18,19]. A rights-based interpretation also requires information on whether children can access inclusive education, communication supports, assistive technology, family support, and participation on an equal basis with others [4,20].

The age-stratified data show a marked difference between the proportions reported for adults aged 15–64 years and those aged ≥65 years (1.4% and 15.1%, respectively). This pattern is descriptive and is compatible with the well-established association between aging and functional difficulty, but the present data cannot determine the contribution of particular diseases, social circumstances, accessibility, or support arrangements. The study therefore does not demonstrate a causal transition across the life course. The findings should therefore not be described as demonstrating a causal ‘life-course transition’ [19,21]. Future analyses with individual-level age-specific data could examine whether the observed age gradient persists after accounting for sex, health conditions, socioeconomic factors, and contextual characteristics.

Similarly, the predominance of congenital, pregnancy-related, and delivery-related categories among children aged 2–4 years and illness among adults represents a distribution of self-reported reasons in the 2023 survey. These categories should not be interpreted as complete causal explanations of disability. A more appropriate interpretation is that the survey attributed the functional difficulties identified to different reasons across age groups. For children, policy implications should extend beyond prevention or risk reduction to include early support, accessible health and developmental services, inclusive education, communication, family support, participation, and dignity [22,23]. For adults, chronic-condition management and rehabilitation may be relevant alongside accessibility, employment inclusion, independent living, and social participation [23].

The regional findings require particular caution. The household-level indicator and the individual-level compositional measure are not equivalent. The household indicator describes the percentage of households represented by GASTAT’s regional household measure. In contrast, the 2023 survey’s 14.8%, 14.7%, and 14.5% figures describe the distribution of severe-or-unable single-disability cases across regions. The latter is necessarily affected by population size. It is therefore appropriate to use Figure 2A to describe regional differences in the published household indicator and Figure 2B to describe where a larger share of cases is located, but not to infer regional risk from Figure 2B.

Regional estimates were not age-standardized. Given the large age difference in disability prevalence, differences in regional age structure may contribute to the observed variation [24]. The aggregate sources used here did not provide the age-specific regional data required for an age-standardized analysis. Additional research using individual-level or sufficiently stratified regional data is needed to determine whether regional differences persist after accounting for age and other demographic factors.

The higher percentage of traffic-accident attribution among males and the different distributions of aging- and illness-related reasons by sex are descriptive findings. Explanations such as greater exposure to road traffic or differences in longevity are plausible but cannot be tested with the available aggregate data. These interpretations should therefore be considered hypotheses for future research rather than established explanations [25,26].

### 4.1. Strengths and Limitations

A strength is the use of recent national GASTAT indicators covering all 13 administrative regions and applying WG-aligned measurement conventions. The study also separates national prevalence, functional multiplicity, household indicators, regional composition, and self-reported reasons rather than treating them as a single outcome. Numerical derivations are explicitly traceable to published proportions and Census denominators, and the manuscript distinguishes descriptive measures from quantities that would require individual-level linkage or inferential analysis.

The principal limitation is the aggregate nature of the available data. No individual-level linkage between the Census and Disability Survey was performed, and no microdata, sampling weights suitable for re-analysis, standard errors, or confidence intervals were publicly available for this study. Consequently, we could not perform inferential testing, multivariable adjustment, intersectional analysis, causal inference, or age-standardized regional comparisons. This was a deliberate restriction, not an attempt to substitute aggregate data for individual-level analysis.

The derived national counts are approximate because they are calculated from published rounded proportions. The Census and Disability Survey also use related but not identical measurement and reporting structures, so their indicators should not be treated as directly interchangeable or as an individually linked longitudinal dataset. The 2023 survey’s reported reasons may be affected by recall, attribution, and reporting error and were not clinically validated. Regional compositional percentages reflect population size and age structure, while household-level regional percentages use different units and denominators. The observed nationality difference may likewise reflect age and sex composition, migration and employment selection, duration of residence, service access, or reporting differences, none of which can be separated with the available aggregate data. Finally, the study does not measure environmental barriers, accessibility, participation restrictions, or unmet need. It therefore cannot quantify disability in the full ICF/CRPD sense or evaluate the effectiveness of specific rehabilitation or policy interventions.

### 4.2. Demographic Selection Mechanics in Non-Saudi Populations

The observed prevalence disparity between non-Saudi residents (1.8% overall; 0.6% severe) and Saudi citizens (5.9% overall; 2.7% severe) may be partly explained by differences in population composition and selection mechanisms rather than by true differential biological protection. These are descriptive population differences, and several forms of population composition and selection could contribute to the observed gap. First, non-Saudi residents in Saudi Arabia have a markedly different age and sex structure from the Saudi population. GASTAT census materials show that non-Saudi residents are strongly concentrated in working-age groups and are disproportionately male, including across several working-age strata [27]. Because the prevalence of operational disability is substantially higher at older ages in the present data, differences in age composition alone could contribute materially to the nationality contrast.

Second, migration and employment selection may contribute. Saudi Arabia has operated an expatriate-worker fitness-screening program involving medical examinations before and upon arrival as part of the worker fitness process [28]. International literature on the healthy immigrant effect indicates that selective migration, screening, and migration-related policies can contribute to healthier observed profiles in some immigrant populations, although the magnitude and direction of these effects vary by population, outcome, and policy context [29,30]. A related healthy-worker selection mechanism may also be relevant where the non-Saudi population is disproportionately composed of economically active migrants. These mechanisms are plausible contextual explanations, but this study did not demonstrate them as causes of the observed difference.

Additional explanations include differences in duration of residence, occupational composition, household structure, health-service access, language and cultural factors, and recognition or reporting of functional difficulty. The available aggregate data do not permit these mechanisms to be separated. In particular, the present study cannot establish that pre-employment screening, workforce selection, or differential reporting caused the lower prevalence, nor can it quantify the contribution of any single mechanism. Individual-level analyses stratified by age, sex, employment, duration of residence, socioeconomic characteristics, and relevant health conditions would be required to evaluate these explanations.

### 4.3. Interpretation of Self-Reported Reasons

Self-reported cause attributions in the GASTAT Disability Survey reflect respondent-perceived onset drivers rather than clinically or longitudinally validated causal diagnoses. These distributions provide descriptive insight into perceived burden and attribution patterns but should not be interpreted as confirmed pathological mechanisms.

The age-patterned distribution of reported reasons is potentially useful for descriptive surveillance but should not be presented as a hierarchy of verified causes. The GASTAT categories reflect respondents’ or household informants’ reported attributions and were not clinically validated against diagnostic records, longitudinal histories, or standardized causal adjudication. The predominance of congenital, pregnancy-related, and delivery-related categories among children aged 2–4 years and illness among adults therefore indicates differences in reported attribution across age groups, not verified differences in causal mechanisms.

For children, the findings may support further investigation of developmental and early-life service needs. Still, they do not establish that prevention of a particular prenatal or perinatal factor would reduce disability prevalence. Appropriate policy interpretation should include early identification, developmental assessment, family support, inclusive education, communication supports, accessibility, participation, and access to rehabilitation where indicated. For adults, chronic-condition management and rehabilitation may be relevant alongside accessibility, employment inclusion, independent living, and social participation. These are potential areas for integrated planning rather than interventions demonstrated by this study.

The findings may inform disability-inclusive planning, but they do not, on their own, establish which service model, allocation formula, or intervention is most appropriate. The study contains no individual-level information on unmet need, service utilization, costs, accessibility, or regional service capacity sufficient to evaluate specific models such as hub-and-spoke rehabilitation. Future planning could use these national descriptive indicators together with evidence on service availability, geographic accessibility, assistive technology, inclusive education, employment, transportation, independent living, anti-discrimination measures, and participation. This broader approach aligns with disability-inclusive policy and the CRPD’s rights-based emphasis.

## 5. Conclusions

The published national indicators provide a useful descriptive baseline for disability and functional difficulty monitoring in Saudi Arabia. The data show marked age-patterned differences in operational disability, substantial multidomain functional difficulty among severe-or-unable cases, and distinct distributions of reported reasons across age groups. Regional case shares describe population distribution rather than comparative regional risk, and the lower prevalence reported among non-Saudi residents should be interpreted in light of differences in age and sex composition and possible migration- and employment-related selection. Reported reasons are respondent-attributed and not clinically verified causes. These findings can support disability-inclusive surveillance and planning, while highlighting the need for individual-level, age-standardized, longitudinal, and contextual research to assess determinants, unmet need, accessibility, and the effectiveness of rehabilitation and other interventions under Saudi Vision 2030.

## Figures and Tables

**Figure 1:**
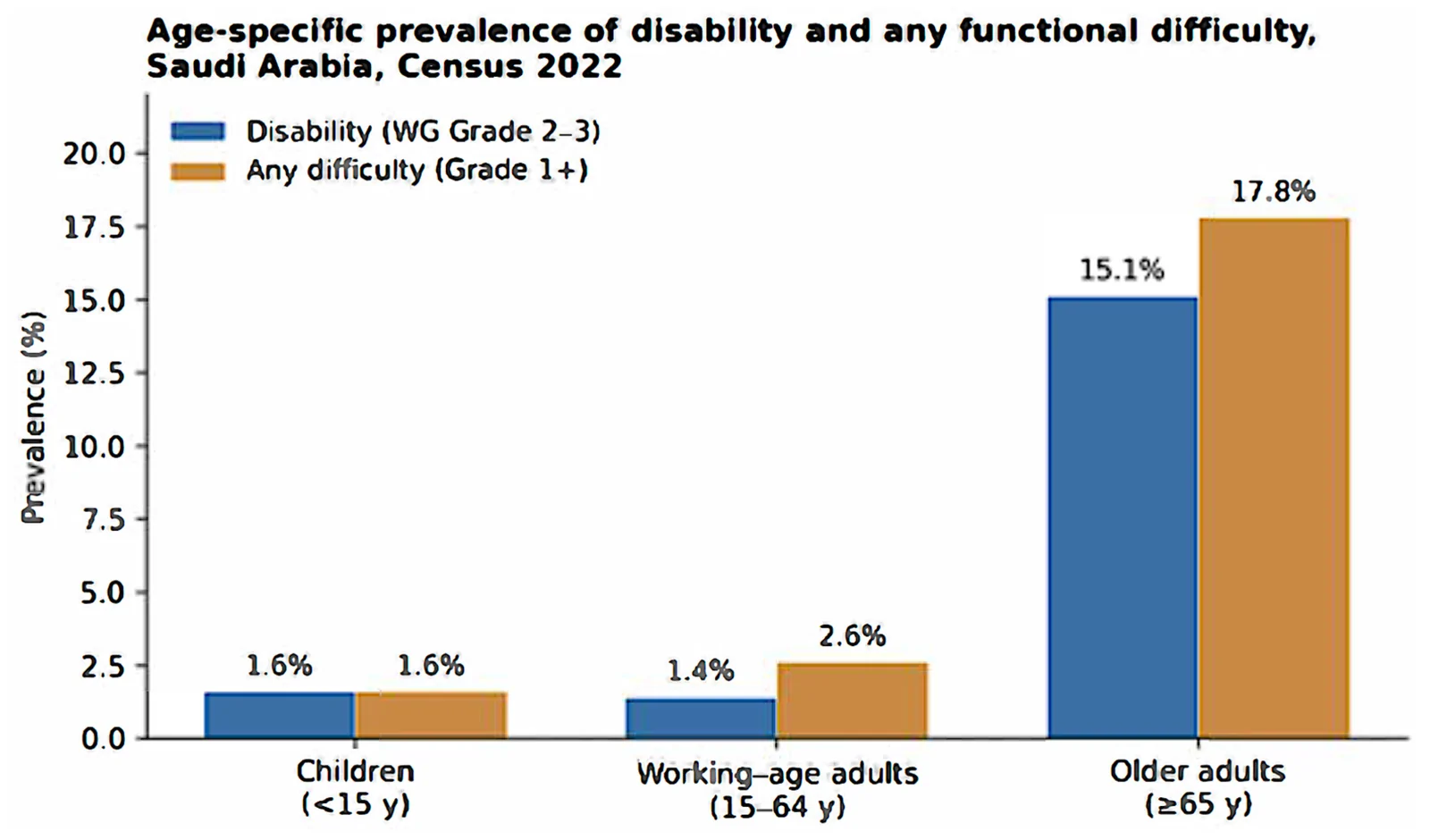
Age-specific prevalence of disability (WG Grades 2–3) and functional difficulty (Grade 1+) in Saudi Arabia: Census 2022. Source: GASTAT Persons with Disability Statistics Report 2023, Indicators 1.3 and 2.3. The figure is descriptive and should not be interpreted as evidence of a causal age effect.

**Figure 2:**
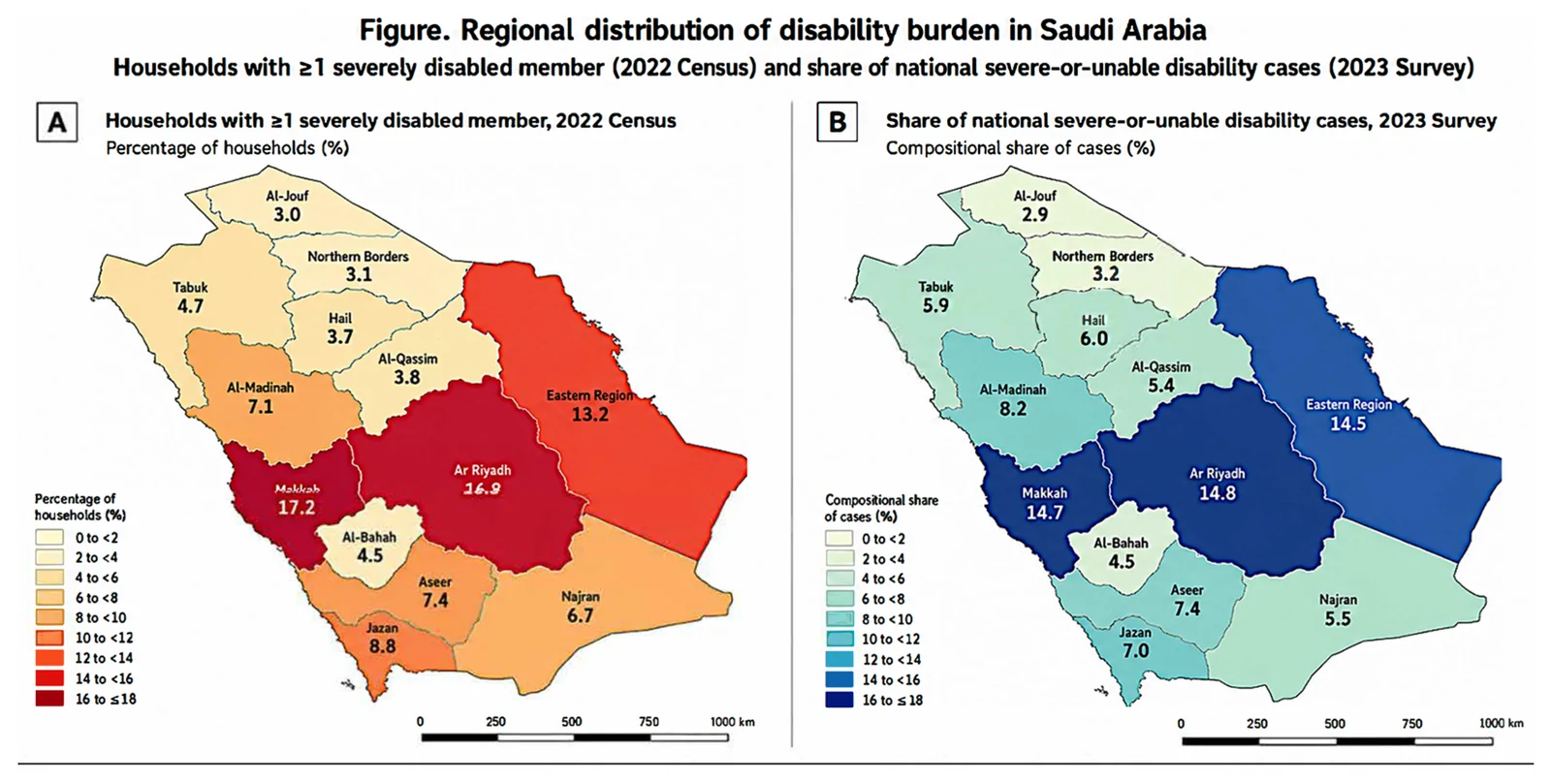
Regional distribution of disability indicators across Saudi Arabia’s 13 administrative regions. (**A**) Percentage of households with ≥1 resident experiencing functional difficulty (GASTAT Census 2022). (**B**) Compositional share of severe-or-unable single-disability cases (2023 Disability Survey). Map callouts and legend boxes are offset to preserve regional boundary legibility.

**Figure 3:**
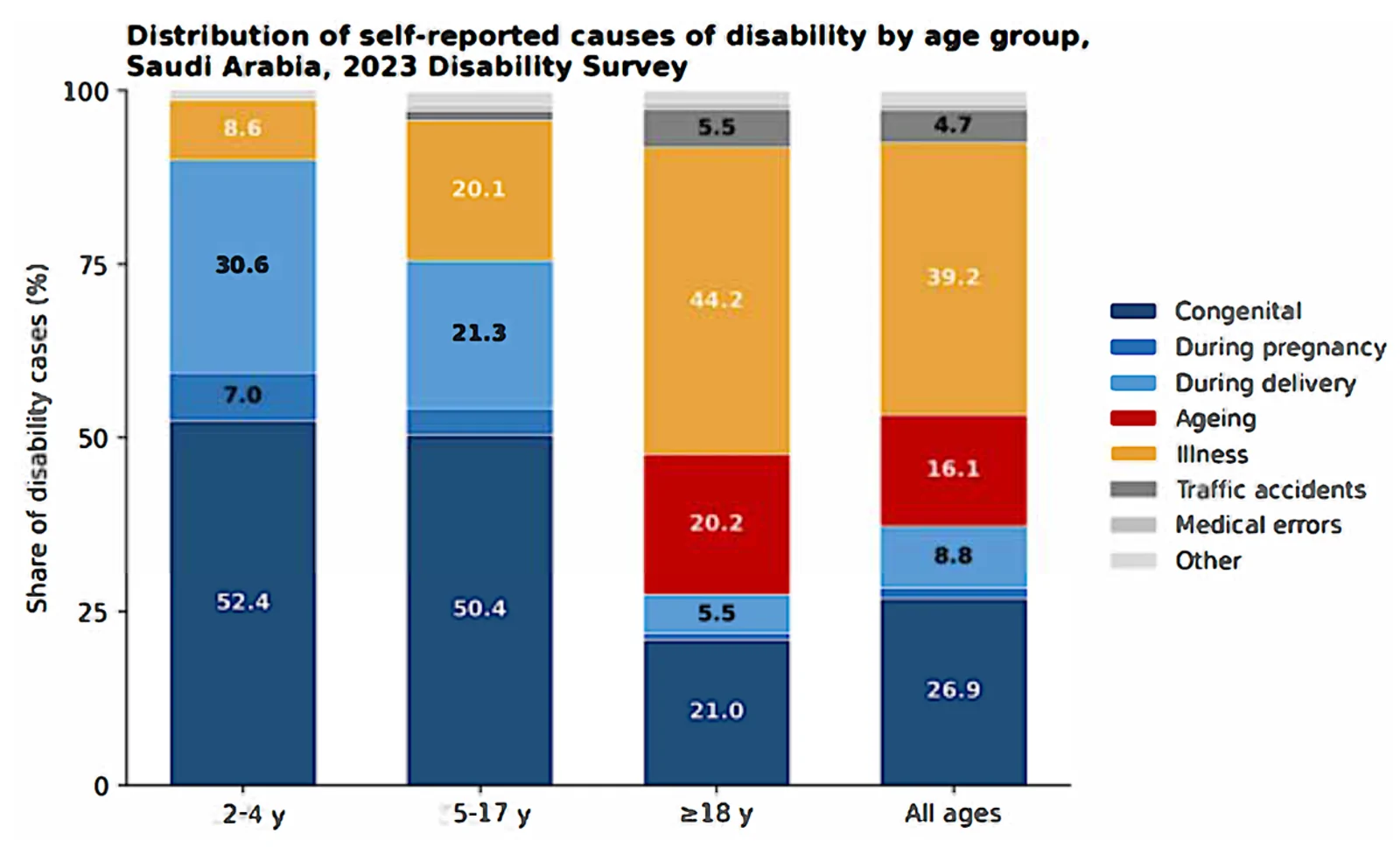
Distribution of self-reported reasons for disability by age group: Saudi Arabia, 2023 Disability Survey. Congenital, pregnancy-related, and delivery-related categories jointly account for 90.0% of reported reasons among children aged 2–4 years; illness and aging together account for 64.4% among adults aged ≥18 years. These are descriptive reported-attribution categories rather than independently verified causes.

**Table 1:** Estimated national prevalence of disability and functional difficulty in Saudi Arabia by nationality, sex, and age group: Census 2022.

Indicator	Group	Prevalence (%)	Population Denominator	Estimated Count
Combined disability + difficulty	Total population	4.2	32,175,200	1,351,400
Combined disability + difficulty	Saudi citizens	5.9	18,803,200	1,109,400
Combined disability + difficulty	Non-Saudi residents	1.8	13,372,000	240,700
Disability (WG Grade 2–3)	Saudi citizens	2.7	18,803,200	507,700
Disability (WG Grade 2–3)	Non-Saudi residents	0.6	13,372,000	80,200
Any difficulty (WG Grade 1+)	Saudi citizens	3.8	18,803,200	714,500
Any difficulty (WG Grade 1+)	Non-Saudi residents	1.3	13,372,000	173,800
Any difficulty by sex	Male	2.6	—	—
Any difficulty by sex	Female	3.0	—	—
Disability by age	Children (<15 y)	1.6	~8,430,000	134,900
Disability by age	Adults (15–64 y)	1.4	~21,590,000	302,300
Disability by age	Older adults (≥65 y)	15.1	~2,156,000	325,500
Any difficulty by age	Children (<15 y)	1.6	~8,430,000	134,900
Any difficulty by age	Adults (15–64 y)	2.6	~21,590,000	561,300
Any difficulty by age	Older adults (≥65 y)	17.8	~2,156,000	383,700

Note: GASTAT reported the prevalence values from Census 2022. We derived nationality-specific estimated counts by multiplying the published prevalence proportions by the corresponding exact Census 2022 population denominators and rounding to the nearest hundred. Standard errors were not publicly released and could not be reconstructed. Age-stratum denominators are approximate and reflect the published Census 2022 age structure. For children aged <15 years, the published aggregate indicators report 1.6% for both the Grade 2–3 and Grade 1+ categories. This identity reflects the rounded aggregate values reported by GASTAT; because the underlying unrounded estimates are not available, identical published percentages should not be interpreted as evidence that no Grade 1-only difficulty occurred. The corresponding derived national approximations are therefore identical when the same published 1.6% is applied to the same age-stratum denominator. WG, Washington Group.

**Table 2:** Distribution of single and functional multimorbidity by age group and sex: Saudi Arabia, 2023 Disability Survey.

Stratum	Single Disability (%)	Functional Multimorbidity (%)
Age 2–4 years	31.8	68.2
Age 5–17 years	40.0	60.0
Age ≥ 18 years	42.0	58.0
Overall (all ages, ≥2 y)	41.5	58.5
Male (severe-or-unable)	45.0	55.0
Female (severe-or-unable)	40.6	59.4
Male (including mild difficulty)	50.8	49.2
Female (including mild difficulty)	49.0	51.0

Note: Source: GASTAT 2023 Disability Survey. Age-stratified percentages exclude mild difficulty. Sex-stratified percentages are shown both excluding and including mild difficulty.

**Table 3:** Regional distribution of disability indicators in Saudi Arabia.

Administrative Region	Households with ≥1 Person with Disability, Including Mild Difficulty (%) [a]	Share of Severe-or-Unable Single-Disability Cases (%) [b]	2016 Prevalence per 100,000 [c]
Ar-Riyadh	14.7	14.8	3437
Makkah	15.0	14.7	3785
Eastern Region	11.7	14.5	3506
Madinah	10.2	8.2	2736
Aseer	7.8	7.4	3641
Jazan	7.5	7.0	3081
Hail	4.2	6.0	2088
Tabuk	6.0	5.9	4308
Najran	5.8	5.5	2898
Al-Qassim	5.4	5.4	2045
Al-Bahah	4.4	4.5	1304
Al-Jouf	3.6	3.2	3786
Northern Borders	3.8	2.9	1339

Note: [a] GASTAT Persons with Disability Statistics Report 2023, Indicator 4.1. The 14.7–3.6% series is the household indicator retained for this analysis. The separate 16.8–3.0% series displayed elsewhere within the published Indicator 4.1 presentation was previously and incorrectly labeled in the submitted manuscript as household prevalence of severe disability. Because the published presentation does not provide sufficient denominator detail to support that interpretation, that series has been removed from the analysis rather than treated as a nested Grade 2–3 measure. [b] GASTAT 2023 Disability Survey, regional aggregate (severe-or-unable single-disability cases only). This is a compositional share of national cases, not regional prevalence or regional risk. [c] Individual-level prevalence per 100,000 reported by Bindawas and Vennu [17] for the 2016 Demographic Survey, presented only as historical context; methodological differences preclude direct comparison with the current household indicator. Rounding note: percentages may not sum to exactly 100% where applicable because the source data were rounded.

**Table 4:** Distribution of self-reported reasons for disability by age group and sex: Saudi Arabia, 2023 Disability Survey.

Reported Reason	2–4 y (%)	5–17 y (%)	≥18 y (%)	Male (%)	Female (%)	Total (%)
Congenital factors	52.4	50.4	21.0	28.0	25.6	26.9
During pregnancy	7.0	3.8	0.9	1.5	1.5	1.5
During delivery	30.6	21.3	5.5	8.9	8.7	8.8
Traffic accidents	0.2	1.4	5.5	7.2	1.7	4.7
Ageing	—	—	20.2	14.2	18.5	16.1
Illness	8.6	20.1	44.2	37.2	41.7	39.2
Medical errors	0.5	0.9	0.9	1.0	0.8	0.9
Other	0.8	1.9	1.8	2.0	1.5	1.8

Note. Source: GASTAT Persons with Disability Statistics Report 2023, Indicator 1.4. These are self-reported or reported-attribution categories and should not be interpreted as independently verified causal effects. Age- and sex-stratified marginals are presented separately because joint cause × age × sex cross-tabulation is not available in the public aggregate release. Percentages may not total exactly 100% because of rounding.

## Data Availability

All data analyzed in this study are publicly available from the General Authority for Statistics, Kingdom of Saudi Arabia. The Persons with Disability Statistics Report 2023 and the Saudi Population and Housing Census 2022 indicator outputs underpinning this analysis are accessible without restriction.

## Supplementary Materials

The following supporting information can be downloaded at: https://www.mdpi.com/article/10.3390/jcm15197504/s1. Supplementary File S1: Extended Methods, RECORD-Extended STROBE Checklist, and Related Tables; Supplemental Box S1: Extended methods; Supplemental Box S2: Note on data access; Supplemental Table S1: RECORD-extended STROBE checklist for the present analysis [31]; Supplemental Table S2: Census 2022 disability and any-difficulty prevalence indicators by nationality, sex, and age group, with applied denominators and derived counts; Supplemental Table S3: Aggregate derivation audit for selected national counts; Supplemental Table S4: 2023 Survey demographic and severity indicators (Indicators 1.1–1.5); Supplemental Table S5: Distribution of functional impairments among individuals with a single severe or unable disability by age cohort, Saudi Arabia, 2023 Disability Survey; Supplemental Table S6: Assistive-technology utilization, residual functional limitation, and reported unmet need among individuals with a single severe or unable disability, Saudi Arabia, 2023 Disability Survey; Supplemental Table S7: Regional distribution from Census 2022 (household level) and 2023 Survey (compositional share), with 2016 reference values from Bindawas and Vennu (2018) [17]; Supplemental Table S8: 2023 Survey cause-of-disability indicators (Indicator 1.4) by age and sex.

## Abbreviations

The following abbreviations are used in this manuscript:

GASTAT	General Authority for Statistics
ICF	International Classification of Functioning, Disability and Health
CRPD	Convention on the Rights of Persons with Disabilities
NCDs	noncommunicable diseases
RECORD	Reporting of studies conducted using observational, routinely collected health Data
SDG	Sustainable Development Goal
STROBE	Strengthening the Reporting of Observational Studies in Epidemiology
UNICEF	United Nations Children’s Fund
WG	Washington Group
WHO	World Health Organization
YLDs	years lived with disability
