# Supplementary material for: The Epidemiology of Disability in Saudi Arabia: National Prevalence, Functional Multimorbidity, and Age-Stratified Self-Reported Causes
Source: J Clin Med. 2026 Sep 27;15(19):7504. doi: 10.3390/jcm15197504 (PMC13666446; doi:10.3390/jcm15197504)
Supplement: Supplementary file 1 [file jcm-15-07504-s001.zip › jcm-4571074-supplementary.pdf]

The Epidemiology of Disability in Saudi Arabia: National Prevalence, Functional Multimorbidity, and Age-Stratified Self-Reported Causes

Supplementary File S1: Extended Methods, RECORD-Extended STROBE Checklist, and Related Tables. Supporting material for: National Burden, Regional Distribution, and Demographic Patterning of Disability in Saudi Arabia: An Epidemiological Analysis of the 2022 Population and Housing Census and the 2023 Disability Survey.

Supplemental Box S1: Extended methods

Source data and aggregation hierarchy

The analysis drew on two officially published GASTAT outputs: the Saudi Population and Housing Census 2022 indicator set, summarized in the Persons with Disability Statistics Report 2023 (Indicators 1.1–1.3 for disability prevalence; 2.1–2.3 for any-difficulty prevalence; 4.1 for regional household-level metrics); and the 2023 Disability Survey aggregate output, including ten public indicator tables covering demographic, functional, occupational, household, and developmental dimensions. The two sources were combined under the Washington Group framework: Census 2022 supplied national, nationality-stratified, sex-stratified, and age-stratified prevalence; the 2023 Survey supplied severity decomposition, multiplicity, cause attribution, regional compositional shares, functional-domain breakdown, and assistive-device context. Microdata were not accessed; all extracted indicators were already in aggregate proportional form at release.

Indicator extraction and auditability

Every indicator used in the present analysis is documented within this supplementary file. Each row records the source publication, the indicator number or table identifier, the page or sheet reference, the published proportion or rate, the denominator (where available), and the derived count (where computed). This document supports an end-to-end audit of every numerical value reported in Tables 1–4 and Figures 1–3 of the main manuscript and the supplemental tables of this document.

Derivation of estimated counts

Estimated absolute counts were derived using the formula: Count = Published Prevalence Proportion × Census 2022 Denominator. The denominators applied were 32,175,224 for the total resident population, 18,803,176 for Saudi citizens, and 13,372,048 for non-Saudi residents. Age-stratum denominators were derived by applying the published Census 2022 age structure (approx. 26.2% under 15 years, approx. 67.1% aged 15–64 years, and approx. 6.7% aged >= 65 years) to the total denominator. All derived counts are rounded to the nearest hundred and are flagged as approximations in Table 1 of the main manuscript and the relevant supplemental tables below.

Two-tier severity convention and departure from three-tier decomposition

The Washington Group convention codes severity along three response levels (Grade 1: mild; Grade 2: severe; Grade 3: very severe or unable). The 2023 GASTAT Survey publishes results using a two-tier dichotomy: Grade 1 mild difficulty (31.8% of identified individuals) versus Grades 2–3 severe or unable disability (68.2%). Nine of ten public 2023 Survey tables exclude Grade 1 entirely. A finer three-tier decomposition is therefore not extractable from the publicly released aggregates, and any analysis dependent on separating Grade 2 from Grade 3 would require access to GASTAT microdata. Severity is reported throughout the present analysis as the published two-tier dichotomy.

Supplemental Box S2: Note on data access

All analysis inputs are publicly available indicator outputs from the General Authority for Statistics, Kingdom of Saudi Arabia, and can be downloaded from https://www.stats.gov.sa. Supplementary File 1 and the tables provide an indicator-level data dictionary linking each figure in the manuscript and supplement to its source, enabling a complete audit of the numerical chain..

Supplemental Table S1: RECORD-extended STROBE checklist for the present analysis.

Item	Recommendation	Location in manuscript	Status
1a	Title indicates study design	Title page	Met

1b	Structured abstract	Abstract (Background/Methods/Results/Conclusions)	Met
2	Background and rationale	Introduction §1 paragraphs 1–4	Met
3	Specific objectives or hypotheses	Introduction §1 paragraph 5	Met
4	Study design (cross-sectional secondary analysis)	Methods §2.1	Met
5	Setting (Kingdom of Saudi Arabia, all 13 administrative regions)	Methods §2.1	Met
6a	Eligibility criteria; sources and methods of selection	Methods §2.1 (households containing residents ≥2 years; n=29,920)	Met
7	Variables clearly defined (disability, functional difficulty, severity)	Methods §2.2	Met
8	Data sources/measurement (WG-ES, UNICEF/WG CFM, abbreviated WG screening)	Methods §2.2	Met
9	Bias addressed (reporting bias, instrument heterogeneity, hard-to-enumerate populations)	Discussion §4.6	Met
10	Study size/sampling frame (29,920 households; Census 2022 frame)	Methods §2.1	Met
11	Quantitative variables and severity convention	Methods §2.2 and §2.3	Met
12a	Statistical methods	Methods §2.4 (descriptive only; counts derived from published proportions)	Met
12b	Subgroup methods	Methods §2.4 (nationality, sex, age stratification)	Met
12c	Methods to address missing data	Methods §2.4 (no microdata accessed; missingness handled at GASTAT level)	Met
12d	Sensitivity analyses	Methods §2.4 (limited by aggregate-only nature; flagged in Discussion §4.6)	Acknowledged limitation
13a	Numbers of individuals at each stage of study	Methods §2.1 (sample frame); Results §3.1 (national counts)	Met
14a	Descriptive characteristics of participants	Results §3.1 and §3.2; Table 1	Met
15	Outcome data (prevalence, severity, multiplicity, cause)	Results §3.1–§3.5; Tables 1–4; Figures 1–3	Met
16	Main results (prevalence with derived counts; regional shares; cause distribution)	Results §3.1–§3.5	Met
17	Other analyses (sex-stratified causes, regional comparison with 2016)	Results §3.4–§3.5; Table 3	Met
18	Key results summarised	Discussion §4 paragraph 1	Met
19	Limitations	Discussion §4.6	Met
20	Interpretation in context	Discussion §4.1–§4.5	Met
21	Generalisability	Discussion §4.6 (national coverage; non-Saudi underestimation caveat)	Met
22	Funding	Funding section (placeholder)	To be completed
—	RECORD-extended item: Description of routinely-collected data sources accessed	Methods §2.1 (GASTAT public indicators)	Met
—	RECORD-extended item: Codes used to identify exposures, outcomes, and confounders	Methods §2.2 (Washington Group definitions)	Met

—	RECORD-extended item: Methods for cleaning routinely-collected data	Methods §2.3 (no cleaning required; aggregate inputs used as published)	Met
—	RECORD-extended item: Linkage of databases	Not applicable (single-source aggregate analysis from GASTAT)	Not applicable
—	RECORD-extended item: Data access and cleaning script availability	Supplementary File S1 (Methods and Supplemental Tables)	Met

Adapted from the STROBE statement (von Elm et al., PLoS Med 2007) and the RECORD extension (Benchimol et al., PLoS Med 2015). The combined checklist supports transparent reporting of observational studies that use officially published, routinely collected aggregate data of the kind released by national statistical agencies.

Supplemental Table S2: Census 2022 disability and any-difficulty prevalence indicators by nationality, sex, and age group, with applied denominators and derived counts

Source	Indicator ID	Page / location	Description	Published value (%)	Denominator applied	Derived count	Notes
GASTAT Persons with Disability Statistics Report 2023	1.1	p. 13	Prevalence of disability and difficulty by nationality (combined)	4.2 (total); 5.9 (Saudi); 1.8 (non-Saudi)	32,175,224 total; 18,803,176 Saudi; 13,372,048 non-Saudi	1,351,400 total; 1,109,400 Saudi; 240,700 non-Saudi	Census denominators from official Census 2022 totals
GASTAT Persons with Disability Statistics Report 2023	1.1 (sub)	p. 13	Prevalence of disability (WG Grade 2–3) by nationality	2.7 (Saudi); 0.6 (non-Saudi)	Saudi 18,803,176; non-Saudi 13,372,048	507,700 (Saudi); 80,200 (non-Saudi); 587,900 combined	
GASTAT Persons with Disability Statistics Report 2023	2.1	p. 14	Prevalence of any difficulty (WG Grade 1+) by nationality	3.8 (Saudi); 1.3 (non-Saudi)	Saudi 18,803,176; non-Saudi 13,372,048	714,500 (Saudi); 173,800 (non-Saudi); 888,400 combined	
GASTAT Persons with Disability Statistics Report 2023	1.2	p. 13	Prevalence of disability by sex (Saudi citizens; published indicator)	1.6 (Saudi); 2.1 (non-Saudi)	Refers to within-nationality sex distribution at the national level	—	Note: GASTAT row labels Saudi/Non-Saudi here despite header 'sex'; interpretation as nationality-level proportion of the disabled subgroup
GASTAT Persons with Disability Statistics	2.2	p. 14	Prevalence of any difficulty by sex	2.6 (male); 3.0 (female)	Total resident population by sex	—	

Report 2023							
GASTAT Persons with Disability Statistics Report 2023	1.3	p. 13	Prevalence of disability by age group	1.6 (children <15 y); 1.4 (15–64 y); 15.1 (≥65 y)	~8.43m (<15 y); ~21.59m (15–64 y); ~2.16m (≥65 y)	134,900; 302,300; 325,500 respectively	Age structure ≈26.2% / 67.1% / 6.7% (Census 2022)
GASTAT Persons with Disability Statistics Report 2023	2.3	p. 14	Prevalence of any difficulty by age group	1.6 (<15 y); 2.6 (15–64 y); 17.8 (≥65 y)	Same as 1.3 above	134,900; 561,300; 383,700	

Supplementary Table S3: Aggregate derivation audit for selected national counts

Indicator	Published prevalence	Census 2022 denominator	Derived count	Calculation
Combined disability + functional difficulty, total population	4.2%	32,175,224	~1,351,400	32,175,224 × 0.042
Combined disability + functional difficulty, Saudi citizens	5.9%	18,803,176	~1,109,400	18,803,176 × 0.059
Combined disability + functional difficulty, non-Saudi residents	1.8%	13,372,048	~240,700	13,372,048 × 0.018
Operational disability (Grade 2–3), Saudi citizens	2.7%	18,803,176	~507,700	18,803,176 × 0.027
Operational disability (Grade 2–3), non-Saudi residents	0.6%	13,372,048	~80,200	13,372,048 × 0.006

Supplemental Table S4: 2023 Survey demographic and severity indicators (Indicators 1.1–1.5)

Source	Indicator ID	Page / location	Description	Published value (%)	Notes
GASTAT 2023 Disability Survey	Preamble	p. 15	Severity dichotomy among identified individuals	31.8 mild (Grade 1); 68.2 severe-or-unable (Grade 2–3)	Two-tier publication convention
**GASTAT 2023 Disability Survey**	Indicator 1 (sex)	p. 16	Single vs multiple disability by sex (excluding mild)	Male: 45.0 single / 55.0 multiple; Female: 40.6 / 59.4	Severe-or-unable cases only
**GASTAT 2023 Disability Survey**	Indicator 1.2	p. 16	Single vs multiple disability by sex (including mild)	Male: 50.8 / 49.2; Female: 49.0 / 51.0	All identified individuals
**GASTAT 2023 Disability Survey**	Indicator 1.3	p. 16	Single vs multiple disability by age group (excluding mild)	2–4 y: 31.8 / 68.2; 5–17 y: 40.0 / 60.0; ≥18 y: 42.0 / 58.0	Severe-or-unable cases only
**GASTAT 2023 Disability Survey**	Derived overall	—	Single vs multiple disability across all severe-or-unable cases (weighted average implied by sample frame)	41.5 single / 58.5 multiple	Approximate weighted from age-stratum data

Supplemental Table S5: Distribution of functional impairments among individuals with a single severe or unable disability by age cohort, Saudi Arabia, 2023 Disability Survey.

Functional domain	2–4 y (%)	5–17 y (%)	≥18 y (%)
Vision	31.6	30.8	19.9
Hearing	10.2	7.6	6.9
Mobility (walking and stair-climbing)	29.0	35.7	56.2
Mobility (upper-body)	—	—	3.8
Movement of hands and fingers	2.8 (fine motor)	—	0.6
Movement of arms	—	—	2.9
Communication	17.5	9.8	1.2
Personal care/self-care	—	7.0	0.04
Learning	3.0	2.1	—
Memorising or remembering	—	0.9	4.7 (memorising and concentrating)
Concentrating	—	0.6	2.9
Accepting change	—	1.3	—
Controlling behaviour	0.0	1.8	—
Making friends	—	1.9	—
Persistent anxiety	—	—	0.6 (depression and anxiety together: 0.5)
Persistent depression	—	—	0.3
Permanent anxiety and depression	—	0.5	—
Playing	6.0	—	—

Source: GASTAT 2023 Disability Survey, Indicator 1 figures (Figures 1–3 in the GASTAT report) [24]. An em-dash indicates the domain is not assessed in the relevant age stratum or is not separately reported. Combined-domain entries reflect the publication format of the source.

Supplemental Table S6: Assistive-technology utilization, residual functional limitation, and reported unmet need among individuals with a single severe or unable disability, Saudi Arabia, 2023 Disability Survey.

Indicator	Value (%)
Use of corrective glasses or contact lenses (any user)	63.4
Use of hearing aids (any user)	10.3
Use of mobility-assistive device or personal mobility assistance (any user)	26.3
Visual impairment persisting despite glasses/lenses (residual)	52.1
Hearing impairment persisting despite hearing aid (residual)	10.9
Mobility impairment persisting despite mobility aid (residual)	37.0
Mobility-aid type—wheelchair (electric or manual)	30.2
Mobility-aid type—walking stick	25.2
Mobility-aid type—crutches	23.5
Mobility-aid type—assistance from another person	16.5
Mobility-aid type—walker	3.5
Mobility-aid type—prosthetic limb	0.9
Mobility-aid type—other	0.3
Rehabilitation-centre enrolment (≥5 y, includes mild difficulty)	20.3
Non-enrolment in rehabilitation centre (≥5 y)	79.7
Reason for non-enrolment—geographic distance	26.7
Reason for non-enrolment—lack of centres	24.4
Reason for non-enrolment—difficulty of movement / lack of transport	14.2
Reason for non-enrolment—lack of financial support	10.4
Reason for non-enrolment—lack of vacancy	9.3
Reason for non-enrolment—other	15.1
Disability-related living costs >25% of family income	27.5
Disability-related living costs 10–25% of family income	32.7
Disability-related living costs <10% of family income	39.8
Principal source of treatment financing—government assistance	73.0

Principal source of treatment financing—family	20.3
Principal source of treatment financing—health insurance	6.0

Source: GASTAT 2023 Disability Survey, Indicators 1.5, 3.2, 3.2.1, and supporting figures [24]. Categories within each domain (mobility-aid type; reasons for non-enrolment) sum to more than 100% where multiple choices were permitted at the respondent level. Detailed analysis of these indicators forms the basis of the companion paper on rehabilitation coverage and assistive-technology adequacy.

Supplemental Table S7: Regional distribution from Census 2022 (household level) and 2023 Survey (compositional share); 2016 reference values from Bindawas and Vennu (2018)

Region	Households ≥1 any difficulty (%) [Census 2022]	Households ≥1 severely disabled (%) [Census 2022]	Compositional share of severe single-disability cases (%) [2023 Survey]	2016 prevalence per 100,000 [Bindawas & Vennu 2018]	Source for 2022 data	Source for 2023 share
Al-Riyadh	14.7	16.8	14.8	3437	GASTAT Report 2023, Indicator 4.1, p. 26	GASTAT 2023 Disability Survey, regional aggregate (Excel)
Makkah	15	17.2	14.7	3785	GASTAT Report 2023, Indicator 4.1, p. 26	GASTAT 2023 Disability Survey, regional aggregate (Excel)
Eastern Region	11.7	13.2	14.5	3506	GASTAT Report 2023, Indicator 4.1, p. 26	GASTAT 2023 Disability Survey, regional aggregate (Excel)
Madinah	10.2	7.1	8.2	2736	GASTAT Report 2023, Indicator 4.1, p. 26	GASTAT 2023 Disability Survey, regional aggregate (Excel)
Aseer	7.8	7.4	7.4	3641	GASTAT Report 2023, Indicator 4.1, p. 26	GASTAT 2023 Disability Survey, regional aggregate (Excel)
Jazan	7.5	8.8	7	3081	GASTAT Report 2023, Indicator 4.1, p. 26	GASTAT 2023 Disability Survey, regional

						aggregate (Excel)
Hail	4.2	3.7	6	2088	GASTAT Report 2023, Indicator 4.1, p. 26	GASTAT 2023 Disability Survey, regional aggregate (Excel)
Tabuk	6	4.7	5.9	4308	GASTAT Report 2023, Indicator 4.1, p. 26	GASTAT 2023 Disability Survey, regional aggregate (Excel)
Najran	5.8	6.7	5.5	2898	GASTAT Report 2023, Indicator 4.1, p. 26	GASTAT 2023 Disability Survey, regional aggregate (Excel)
Al-Qassim	5.4	3.8	5.4	2045	GASTAT Report 2023, Indicator 4.1, p. 26	GASTAT 2023 Disability Survey, regional aggregate (Excel)
Al-Bahah	4.4	4.5	4.5	1304	GASTAT Report 2023, Indicator 4.1, p. 26	GASTAT 2023 Disability Survey, regional aggregate (Excel)
Al-Jouf	3.6	3.1	3.2	3786	GASTAT Report 2023, Indicator 4.1, p. 26	GASTAT 2023 Disability Survey, regional aggregate (Excel)
Northern Borders	3.8	3	2.9	1339	GASTAT Report 2023, Indicator 4.1, p. 26	GASTAT 2023 Disability Survey, regional aggregate (Excel)

Supplemental Table S8: 2023 Survey cause-of-disability indicators (Indicator 1.4) by age and sex

Source	Indicator ID	Page / location	Stratum	Cause of disability	Published value (%)
GASTAT 2023 Disability Survey	1.4	p. 17	2–4 y	Congenital factors	52.4
GASTAT 2023 Disability Survey	1.4	p. 17	2–4 y	During pregnancy	7.0
GASTAT 2023 Disability Survey	1.4	p. 17	2–4 y	During delivery	30.6
GASTAT 2023 Disability Survey	1.4	p. 17	2–4 y	Traffic accidents	0.2
GASTAT 2023 Disability Survey	1.4	p. 17	2–4 y	Ageing	—
GASTAT 2023 Disability Survey	1.4	p. 17	2–4 y	Illness	8.6
GASTAT 2023 Disability Survey	1.4	p. 17	2–4 y	Medical errors	0.5
GASTAT 2023 Disability Survey	1.4	p. 17	2–4 y	Other	0.8
GASTAT 2023 Disability Survey	1.4	p. 17	5–17 y	Congenital	50.4
GASTAT 2023 Disability Survey	1.4	p. 17	5–17 y	During pregnancy	3.8
GASTAT 2023 Disability Survey	1.4	p. 17	5–17 y	During delivery	21.3
GASTAT 2023 Disability Survey	1.4	p. 17	5–17 y	Traffic accidents	1.4
GASTAT 2023 Disability Survey	1.4	p. 17	5–17 y	Illness	20.1
GASTAT 2023 Disability Survey	1.4	p. 17	5–17 y	Medical errors	0.9
GASTAT 2023 Disability Survey	1.4	p. 17	5–17 y	Other	1.9
GASTAT 2023 Disability Survey	1.4	p. 17	≥18 y	Congenital	21.0
GASTAT 2023 Disability Survey	1.4	p. 17	≥18 y	During pregnancy	0.9
GASTAT 2023 Disability Survey	1.4	p. 17	≥18 y	During delivery	5.5
GASTAT 2023 Disability Survey	1.4	p. 17	≥18 y	Traffic accidents	5.5
GASTAT 2023 Disability Survey	1.4	p. 17	≥18 y	Ageing	20.2
GASTAT 2023 Disability Survey	1.4	p. 17	≥18 y	Illness	44.2
GASTAT 2023 Disability Survey	1.4	p. 17	≥18 y	Medical errors	0.9
GASTAT 2023 Disability Survey	1.4	p. 17	≥18 y	Other	1.8
GASTAT 2023 Disability Survey	1.4 (Total)	p. 17	All ages	Congenital	26.9
GASTAT 2023 Disability Survey	1.4 (Total)	p. 17	All ages	During pregnancy	1.5
GASTAT 2023 Disability Survey	1.4 (Total)	p. 17	All ages	During delivery	8.8

GASTAT 2023 Disability Survey	1.4 (Total)	p. 17	All ages	Traffic accidents	4.7
GASTAT 2023 Disability Survey	1.4 (Total)	p. 17	All ages	Ageing	16.1
GASTAT 2023 Disability Survey	1.4 (Total)	p. 17	All ages	Illness	39.2
GASTAT 2023 Disability Survey	1.4 (Total)	p. 17	All ages	Medical errors	0.9
GASTAT 2023 Disability Survey	1.4 (Total)	p. 17	All ages	Other	1.8
GASTAT 2023 Disability Survey – Excel aggregate	Sex breakdown	Excel sheet (cause × sex)	Male / Female	Traffic accidents	Male 7.2; Female 1.7
GASTAT 2023 Disability Survey – Excel aggregate	Sex breakdown	Excel sheet	Male / Female	Illness	Male 37.2; Female 41.7
GASTAT 2023 Disability Survey – Excel aggregate	Sex breakdown	Excel sheet	Male / Female	Ageing	Male 14.2; Female 18.5
